# Migration, Schizophrenia, and Crime: A Study From a Forensic Psychiatric Sample

**DOI:** 10.3389/fpsyt.2022.869978

**Published:** 2022-05-06

**Authors:** Yong He, Yan Gu, Shujian Wang, Yan Li, Gangqin Li, Zeqing Hu

**Affiliations:** ^1^Department of Forensic Psychiatry, West China School of Basic Medical Sciences and Forensic Medicine, Sichuan University, Sichuan, China; ^2^Shanghai Key Laboratory of Forensic Medicine, Key Lab of Forensic Science, Ministry of Justice, Shanghai Forensic Service Platform, Academy of Forensic Science, Shanghai, China

**Keywords:** schizophrenia, internal migrant, demographic characteristics, clinical characteristics, criminological characteristics

## Abstract

**Background:**

The association between mental health problems and crime in immigrants has attracted recent academic interest, with results suggesting that there were possible interactions between immigration, schizophrenia, and criminal behavior. However, very few studies have examined these interactions, especially in developing countries that have mass internal immigration. Therefore, this study sought to identify the associations between the sociodemographic, clinical, and criminal factors in migrants and non-migrants with schizophrenia who had been involved in criminal activities in China.

**Methods:**

Forensic archives of suspects who had been referred for criminal responsibility assessments in the Sichuan West China Forensic Center from January 2015 to December 2019 were reviewed. The sociodemographic, and criminal activity information of the suspects were extracted, while the clinical and social function were measured by the Brief Psychiatric Rating Scale (BPRS) and Social Disability Screening Schedule (SDSS) based on the archives. A Chi-squared test, a T-test, a Mann-Whitney U test, and Multinomial logistic regression were employed for the statistical analysis.

**Results:**

A total of 552 patients were reviewed and evaluated, 17.2% (*n* = 95) of which were migrants. The migrant patient group was younger than the non-migrant patient group. The BPRS and SDSS scores for the migrant patient group were lower than for the non-migrant patient group. The migrant patient group had more work experience and more had been employed at the time of the crime than the non-migrant patient group. The unemployed migrant patients were more likely to commit a property-related crime.

**Conclusions:**

Compared to the non-migrant schizophrenia patient group, the migrant patient group had less severe psychiatric symptoms and less social function impairments. Employment was an important factor in preventing criminality in patients with schizophrenia, especially for migrant patients. Vocational rehabilitation focuses on developing appropriate employment that can significantly assist in schizophrenia patient recovery, which in turn could reduce their chances of committing crime. Besides, reducing other obstacles to stigma, housing and health insurance would also be beneficial to crime reduction.

## Introduction

The association between mental health problems and crime in immigrants has attracted recent academic interest, with the results suggesting that there were possible interactions between immigration, schizophrenia, and criminal behavior. While people generally migrate for social, economic, or political reasons, many studies have found that migration increases the risk of developing schizophrenia (1–4). Furthermore, social conditions, hostility, threats, and violence were found to be the primary determinants for psychotic disorders in migrants (5), In contrast, racial discrimination, social adversity, unemployment, family dysfunction, and poor housing conditions were recognized as contributing factors (4). Besides the increased risk of mental problems, there have also been widespread concerns that immigration increases crime rates; however, studies on the links between immigration and crime have not yielded consistent conclusions. A Swedish study found that 58% of total crime suspects were migrants, and 70% of robbery cases and 73% of murder, manslaughter, and attempted murder cases involved migrants. In particular, from 2002 to 2017, migrants were blamed for the quadrupling of the Swedish murder rate (6). However, in Italy, immigration was only found to be associated with an increase in robbery (7). Bell et al. investigated two large immigration waves in the U.K. (asylum seekers in the late 1990s/early 2000s and the post-2004 inflow from European Union accession countries), and found no significant relationships between immigrants and violent crime, which suggested that the differences in the labor market opportunities for different migrant groups influenced the potential impact on crime (8).

The associations between mental disorders and criminal violence have been extensively studied, especially for people with schizophrenia (9–11). A recent review found that the relative risks of violent outcomes in people with the most diagnosed psychiatric disorders were generally higher than in people without psychiatric disorders, with the total violent crime rates over 5–10 years being 6–10% higher in people with personality and schizophrenia and more than 10% higher in people with substance abuse problems (11). In British, Canadian, and Danish samples, migrants that referred for forensic assessments were more likely to be diagnosed with schizophrenia and less likely to be diagnosed with personality disorders than patients without a migration background (12–14).

Given these associations between immigration and schizophrenia, and the association between immigration and crime, it is plausible to assume that there could be some interactions between immigration, schizophrenia, and criminality. However, very few studies have focused on this possibility, especially in developing countries that experience mass internal immigration (migration across cities or provinces within China). With a population exceeding 1.4 billion, China is the largest developing country in the world. The 2020 Chinese population census showed that there was a floating population of 375.82 million people, of which 124.84 million people had moved to other provinces, and 250.98 million had moved within their provinces (15). The internal migration has given rise to significant social challenges, including mental health and criminality problems (16–18). Floating people with schizophrenia, with functional impairments in cognition, daily life, and vocational and social skills, may pose great health and security problems both for the patients and the public. Research into the characteristics and risk factors of schizophrenic migrants involved in criminality could aid us in managing this kind of population more scientifically, which could be beneficial to increasing public security and decreasing the health and judicial cost of our country.

This study analyzed a sample of internal immigrant forensic psychiatric patients and compared their demographic, clinical, and criminal characteristics with non-immigrant forensic patients. The migrants were divided into two subgroups; those that had moved between provinces (inter-provincial migrants) and those that had moved within one province (within-provincial migrants); and the demographic, clinical, and criminal characteristics again were compared. Rural-to-urban and city-to-urban migration groups were also compared.

## Methods

### Study Subjects

The archives of criminal suspects who had been referred for criminal responsibility assessments and diagnosed with schizophrenia in the Sichuan West China Forensic Center from January 2015 to December 2019 were retrospectively reviewed. The archives included comprehensive demographic, criminal, mental status, and medical history information. Demographic information; gender, ethnicity, year of birth, education level, marital status, place of residence, domicile place, employment history, living situation; clinical information; mental status, drug use, and medical history; and crime information; crime location, crime type, relationship with the victims, injury outcomes, tools used, and criminal history; were collected for the analysis. Schizophrenia was diagnosed based on the criteria in the third edition of the Chinese Classification of Mental Disorders (CCMD-3), which was modeled on the tenth edition of the International Classification of Diseases (19, 20). The Ethics Committee of Sichuan University approved this study.

China's household registration (Hukou) system registers each citizen at a specific place (usually their birthplace) and defines household status by residence location (a specific city, town, or township) and type (either rural or urban) (21). A person's Hukou status affects their social benefits in China, such as access to health care, local medical services, and social security. The floating population identified in the 2020 Chinese population census were all people who had been absent from their registered city for more than 6 months. In this study schizophrenia patients who had committed a crime in a city different from their registered city were classified as migration patients.

### Measurements

#### Brief Psychiatric Rating Scale (BPRS)

The BPRS is an 18-item scale, which has been widely used to measure several symptoms (22). The BPRS has five subscales: affect (anxiety, guilt, depression, somatic); positive symptoms (grandiosity, unusual thoughts, hallucinatory behavioral content, conceptual disorganization); negative symptoms (motor retardation, blunted affect, emotional withdrawal); resistance (suspiciousness, hostility, uncooperativeness); and activation (tension, excitement, mannerisms, and posturing) (23). Total scores range from 18 to 126, with higher scores indicating greater symptom severity (24). The reliability for the Chinese version of BPRS among the Chinese sample is 85 ~ 99% (25). In this study, the BPRS was retrospectively used to evaluate the psychiatric symptoms of patients according to the archives.

#### Social Disability Screening Schedule (SDSS)

The Social Disability Screening Schedule (SDSS) used in this study was a simplified Chinese version of the 1988 World Health Organization's Disability Assessment Schedule, which subjectively measures adult social, occupational, and psychological functioning. The SDSS has 10 items, each of which has a score ranging from zero to two. An epidemiological survey on people with mental disorders in China concluded that a total score of ≥2 points indicated obvious social functioning impairments (26). The reliability for the Chinese version of SDSS among the Chinese sample is 85 ~ 90% (27). In this study the SDSS was retrospectively used to evaluate the social function of patients according to the archives.

### Statistical Analysis

All analyses were performed using IBM SPSS Statistics (version 20.0), with the significance level set at *P* < 0.05 (two-sided). A chi-squared test was used to compare the categorical variables between the groups, the two independent groups were compared using a *T*-test or a Mann-Whitney U test, Multinomial logistic regression was used to examine the effects of employment on criminal type in the migrant and non-migrant groups, and odds ratios revealed the probability of membership in each class compared to the reference class.

## Results

### Demographic Characteristics of the Patients With Schizophrenia

Table 1 gives the demographic details for the 552 patients that were reviewed (81.5% males, 96.2% Chinese Han ethnicity). As can be seen, most were middle-aged (average age of 39.38 ± 11.872 years old) rural residents (70.7%) with ≤ 9 years of formal schooling (78.0%), and most were unmarried, divorced, and widowed (65.6%). While a majority had a history of employment (78.3%), only around one-fifth were employed at the time of their crime. Most of the patients were lived with relatives (72.5%) and only a small percentage (17.2%) were migrants. Of the migrant sample, about 73 percent (72.6%) were within-province migrants and 63% migrated from rural areas to urban areas. In addition, the vast majority (84%) of the migrant patients had suffered schizophrenic episodes before the current migration.

**Table 1:** Comparison of migrant and non-migrant demographic, clinical and criminal characteristics.

	**Migrant**	**Non-migrant**	**Total**	**t/x^**2**^**	***p***
	***N* = 95**	***N* = 457**			
**Demographic characteristics**
Age [Mean (SD)]	35.58 (9.893)	40.18 (12.103)	39.38 (11.872)	−3.468	0.001
**Gender**
Male	84 (88.4%)	366 (80.1%)	450 (81.5%)	3.626	0.057
Female	11 (11.6%)	91 (19.9%)	102 (18.5%)		
**Ethnicity**
Han	92 (96.8%)	439 (96.1%)	531 (96.2%)	–	1.000✰
Others	3 (3.2%)	18 (3.9%)	21 (3.8%)		
**Education years#**
≤ 9	72 (79.1%)	349 (77.7%)	421 (78.0%)	0.085	0.770
>9	19 (20.9%)	100 (22.3%)	119 (22.0%)		
**Household type**
Rural	68 (71.6%)	322 (70.5%)	390 (70.7%)	0.048	0.827
Urban	27 (28.4%)	135 (29.5%)	162 (29.3%)		
**History of employment**
Yes	86 (90.5%)	346 (75.7%)	432 (78.3%)	10.147	0.001
No	9 (9.5%)	111 (24.3%)	120 (21.7%)		
**Employment status**
Employed	42 (44.2%)	78 (17.1%)	120 (21.7%)	34.058	<0.001
Unemployed	53 (55.8%)	379 (82.9%)	432 (78.3%)		
**Marital status**
Married	34 (35.8%)	156 (34.1%)	190 (34.4%)	0.095	0.758
OthersΔ	61 (64.2%)	301 (65.9%)	362 (65.6%)		
**Living situation**
Living with relatives	59 (62.1%)	341 (74.6%)	400(72.5%)	11.527	0.003
Living alone	27 (28.4%)	103 (22.5%)	130 (23.6%)		
Living with others	9 (9.5%)	13 (2.8%)	22 (4.0%)		
**Clinical characteristics**
Age of onset [Mean (SD)] °	28.63 (10.295)	29.86 (11.329)	29.63(11.146)	−0.953	0.341
**History of psychiatric treatment**
Yes	73 (76.8%)	348 (76.1%)	421 (76.3%)	0.021	0.885
No	22 (23.2%)	109 (23.9%)	131 (23.7%)		
**Hospitalization**
Yes	36 (37.9%)	183 (40.0%)	219 (39.7%)	0.152	0.697
No	59 (62.1%)	274 (60.0%)	333 (60.3%)		
**Illness duration(years)**※
<5	40 (42.1%)	139 (30.5%)	179 (32.5%)	4.842	0.028
≥5	55 (57.9%)	317 (69.5%)	372 (67.5%)		
**Medication at the time of violence**
Yes	27 (28.4%)	99 (21.7%)	126 (22.8%)	2.039	0.153
No	68 (71.6%)	358(78.3%)	426 (77.2%)		
**Criminal characteristics**
**History of criminal offense**
Yes	16 (16.8%)	54 (11.8%)	70 (12.7%)	1.794	0.180
No	79 (83.2%)	403 (88.2%)	482 (87.3%)		
**Type of offense**
Violence against a person	45 (47.4%)	317 (69.4%)	362 (65.6%)	21.433	<0.001
Property related	26 (27.4%)	52 (11.4%)	78 (14.1%)		
Others	24 (25.3%)	88 (19.3%)	112 (20.3%)		
**Offense location**
Rural	8 (8.4%)	280 (61.3%)	288 (52.2%)	88.032	<0.001
Urban	87 (91.6%)	177 (38.7%)	264 (47.8%)		

*SD, Standard Deviation; ⋆, Fisher exact test; Δ,Others, unmarried, divorced, widowed; ※ Data were missed from one person in the non-migrant group(n = 551); #Data were missed from four people in the migrant group and eight people in the non-migrant group(n=540); ° Data were missed from three people in the migrant group and forty-eight in the non-migrant group(n = 501)*.

### Clinical Characteristics of the Patients With Schizophrenia

Table 1 shows the clinical characteristic details. The mean age for the schizophrenia onset was 29.63 ± 11.146 years; therefore, a majority (67.5%) had been suffering from the illness for more than 5 years. Over three-quarters of the patients had a history of psychiatric treatment, with 39.7% having been hospitalized in the past. However, regardless of their condition, 77.2% did not take medication at the time of their crime.

### Criminal Characteristics of the Patients With Schizophrenia

As shown in Table 1, over half the criminal acts occurred in rural areas (52.2%). There were only around 12.7% of patients had a criminal history. A majority of the crimes (65.6%) were violent crimes against a person, such as murder, assault, rape, and indecency; 14.1% were property-related crimes, such as theft, robbery, and fraud; while 20.3% were other crimes, such as traffic accidents, damage to property, and arson.

### Comparison Between Migrant and Non-migrant Patients

The migrant and non-migrant patient variables were compared using a chi-squared test (see Table 1). No statistically significant differences were found between the groups for household registration types (*p* = 0.827), ethnicity *(p* = 1.00), education (*p* = 0.770), or marital status (*p* = 0.758). The migrant group patients had a higher proportion of males than the non-migrant group (88.4 vs. 80.1%; *p* = 0.057), and the mean age of the migrant group (35.58 ± 9.893) was significantly lower than the mean age of the non-migrant group (40.18 ± 12.103) (*p* = 0.001). The migrant group patients were less likely to live with relatives (62.1%) than non-migrant group patients (74.6%) (*p* = 0.003). Significantly more migrant group patients had a history of employment (90.5%) than the non-migrant group (75.7%)(*p* = 0.001); in the meanwhile, there were much more (around 44.2%) migrant patients employed at the time of their crime than the non-migrant patients (17.1%)(p <0.001).

As shown in Table 1, significantly (*p* = 0.028) more non-migrant group patients (69.5%) had had schizophrenia for more than 5 years than the migrant group (57.9%). However, there were no significant differences between the migrant and non-migrant patients in terms of antipsychotic treatment histories, hospitalizations, or medication at the time of the crime. The BPRS indicated that there were no significant differences in the affect, activation, or resistance between the groups (*p* > 0.05); however, statistically significant differences were observed in the negative symptoms (*U* = 24927.50, *p* = 0.021), positive symptoms (*U* = 24622.00, *p* = 0.039), and the total scores (*U* = 24697.50, *p* = 0.034), with the median of negative symptoms (5.00 vs. 6.00), positive symptoms (9.00 vs. 12.00), and total BPRS scores (39.00 vs. 42.00) being significantly higher in the non-migrant group. Moreover, the median of SDSS in the non-migrant group patients was 8.00, which was also significantly higher than the score among migrant patients (6.00) (*U* = 26414.500, *P* = 0.001) (see Table 2).

**Table 2:** Comparison of the SDSS and BPRS between the migrant and non-migrant patients.

	**Migrant**	**Non-migrant**	**U※**	***p***
	***N* = 95**	***N* = 457**		
SDSS	6.00 (3.00, 9.00)	8.00 (5.00, 11.00)	26414.50	0.001
Affect	9.00 (7.00, 10.00)	9.00 (7.00, 10.00)	22100.00	0.779
Positive	9.00 (8.00, 14.00)	12.00 (8.00, 15.00)	24622.00	0.039
Negative	5.00 (4.00, 6.00)	6.00 (4.00, 7.00)	24927.50	0.021
Activation	5.00 (3.00, 6.00)	5.00 (4.00, 6.00)	22882.00	0.398
Resistance	8.00 (6.00, 10.00)	9.00 (7.00, 11.00)	24157.50	0.082
BPRS-total	39.00 (33.00, 48.00)	42.00 (34.50, 49.00)	24697.50	0.034

*※Mann-Whitney U test. Numbers outside the brackets are medians of the assessment scores, and those in the brackets are 25% and 75% values*.

As shown in Table 1, the incidence of violent crime against a person was higher in the non-migrant patient group (69.4%) than in the migrant patient group (47.4%); however, the incidence of property-related crimes was significantly higher in the migrant group (27.4%) than in the non-migrant group (11.4%) (*p* < 0.001). No significant differences were observed between the groups for criminal offense history. When the sample was restricted to only include violent crimes against a person, there was a significant difference between the patient victim relationships(*p* < 0.001) between the two groups, with most victims in the migrant group being strangers (60.0%) and most victims in the non-migrant group being acquaintances (51.7%). There was a significant difference (*p* = 0.031) between the two groups in the crime location, with the migrant group committing more crimes in a public space (66.7%) than the non-migrant group (49.2%). There were no statistically significant differences in the number of victims between the two groups; however, much more cases (111 cases, 35.0%) in the non-migrant group have at least one victim death relative to that in the migrant group (nine cases, 20%; *p* = 0.045). The non-migrant group (79.5%) also tended to use more tools in their crimes than the migrant group (66.7%); however, this difference was not statistically significant (*p* = 0.052) (see Table 3).

**Table 3:** Comparison of criminal characteristics between the migrant and non-migrant patients for violent crime against a person.

	**Migrant *N* = 45**	**Non-migrant *N* = 317**	**Total**	**x^**2**^**	***p***
**Location of violent crime against a person**					
Co-residence	8 (17.8%)	70 (22.1%)	78 (21.5%)	9.936	0.031✰
Residence of offender	0 (0.0%)	23 (7.3%)	23 (6.4%)		
Residence of victim	4 (8.9%)	59(18.6%)	63 (17.4%)		
Public place	30 (66.7%)	156 (49.2%)	186 (51.4%)		
Remote place	3 (6.7%)	9 (2.8%)	12 (3.3%)		
**Use of tools**					
Yes	30 (66.7%)	252 (79.5%)	282 (77.9%)	3.767	0.052
No	15 (33.3%)	65 (20.5%)	80 (22.1%)		
**Victim characteristics**					
**Relationship with victims**※					
Relatives	5 (11.1%)	92 (29.0%)	97 (26.8%)	35.852	<0.001
Acquaintances	13 (28.9%)	164 (51.7%)	177 (48.9%)		
Strangers	27 (60.0%)	61 (19.2%)	88 (24.3%)		
**Numbers of victims**					
One	40(88.9%)	267 (84.2%)	307 (84.8%)	0.665	0.415
More than one	5 (11.1%)	50 (15.8%)	55 (15.2%)		
**Gender**					
Male	21 (46.7%)	174 (54.9%)	195 (53.9%)	1.072	0.300
With Female	24 (53.3%)	143(45.1%)	167 (46.1%)		
**Death**					
Yes	9 (20.0%)	111 (35.0%)	120 (33.1%)	4.009	0.045
No	36 (80.0%)	206 (65.0%)	242 (66.9%)		

*✰, Fisher exact test; ※, If there were multiple victims in one case, the relationship was based on the first victim*.

As shown in Table 4, the unemployed migrant patients were more likely to commit property-related crimes (OR = 7.612, 95%CI 2.409–24.055, *p* = 0.001) and other crimes (traffic accident, damage of property, arson, etc.) (OR = 1.320, 95%CI 1.274–10.311, *p* = 0.016) than the employed migrant group patients; however, this difference was not statistically significant in the non-migrant group(p > 0.05).

**Table 4:** Multinomial logistic regression analysis of the effect of employment on criminal type in the migrant and non-migrant groups.

**Groups**	**Variable**	**Property Versus Violence**			**Others Versus Violence**		
		**OR**	**95%CI**	***p***	**OR**	**95%CI**	***p***
Migrant	Unemployed	7.612	2.409–24.055	0.001	1.320	1.274–10.311	0.016
	Employed	reference					
Non-migrant	Unemployed	1.320	0.565–3.083	0.521	0.858	0.468–1.570	0.618
	Employed	reference					

*OR, odds ratio; CI, confidence interval*.

As shown in Tables 5–8, the migrant group was divided into interprovincial migrants and within-province migrants (Table 5) and the sociodemographic, clinical, and criminal features were compared between the two subgroups. The analysis revealed that compared to the interprovincial migrant patients, within-province migrant patients were more likely to have a history of hospitalization(*p* = 0.049) and more likely to offend in urban areas(*p* = 0.005). No statistical differences were found between the two subgroups for any other features (*p* > 0.05). Moreover, no significant difference in sociodemographic, clinical, and criminal features was found between the rural-to-urban migrants and the city-to-urban migrants; however, a significantly higher proportion of the city-to-urban migrants had higher education levels (>9 years) than the rural-to-urban migrants (52.0 vs. 8.6%, respectively; *p* < 0.001).

**Table 5:** Comparison of demographic, clinical and criminal characteristics between inter-province migrant and within province migrant patients.

	**Inter-province migrant**	**Within-province migrant**	**Total**	**t/x^**2**^**	***p***
	***N* = 26**	***N* = 69**			
**Demographic Characteristics**					
Age [Mean (SD)]	34.00 (8.899)	36.17 (10.240)	35.58(9.893)	−0.955	0.342
**Gender**					
Male	22 (84.6%)	62 (89.9%)	84 (88.4%)	–	0.486✰
Female	4 (15.4%)	7 (10.1%)	11 (11.6%)		
**Ethnicity**					
Han	24 (92.3%)	68 (98.6%)	92 (96.8%)	–	0.181✰
Others	2 (7.7%)	1 (1.4%)	3 (3.2%)		
**Education Years#**					
≤ 9	18 (69.2%)	54 (83.1%)	72 (79.1%)	2.155	0.142
>9	8 (30.8%)	11 (16.9%)	19 (20.9%)		
**Household Type**					
Rural	16 (61.5%)	52 (75.4%)	68 (71.6%)	1.774	0.183
Urban	10 (38.5%)	17 (24.6%)	27 (28.4%)		
**History of employment**					
Yes	23 (88.5%)	63 (91.3%)	86 (90.5%)	–	0.702✰
No	3 (11.5%)	6 (8.7%)	9 (9.5%)		
**Employment Status**					
Employed	11 (42.3%)	31 (44.9%)	42 (44.2%)	0.053	0.819
Unemployed	15 (57.7%)	38 (55.1%)	53 (55.8%)		
**Marital Status**					
Married	8(30.8%)	26(37.7%)	34(35.8%)	0.393	0.531
OthersΔ	18(69.2%)	43(62.3%)	61(64.2%)		
**Living Situation**					
Living With Relatives	16 (61.5%)	43 (62.3%)	59(62.1%)	1.682	0.431
Living Alone	9 (34.6%)	18 (26.1%)	27 (28.4%)		
Living With Others	1 (3.8%)	8 (11.6%)	9 (9.5%)		
**Clinical Characteristics**					
Age Of Onset [Mean (SD)]°	29.04 (9.792)	28.47 (10.556)	28.63 (10.295)	0.237	0.813
**History Of Psychiatric Treatment**					
Yes	20 (76.9%)	53 (76.8%)	73 (76.8%)	0.00	0.991
No	6 (23.1%)	16 (23.2%)	22 (23.2%)		
**Hospitalization**					
Yes	12 (46.2%)	47 (68.1%)	59 (62.1%)	3.870	0.049
No	14 (53.8%)	22 (31.9%)	36 (37.9%)		
**Illness Duration (Years)**					
<5	15 (57.7%)	25 (36.2%)	40 (42.1%)	3.568	0.059
≥5	11 (42.3%)	44 (63.8%)	55 (57.9%)		
**Medication at the time of offense**					
Yes	18 (69.2%)	50 (72.5%)	68 (71.6%)	0.097	0.755
No	8 (30.8%)	19 (27.5%)	27 (28.4%)		
**Onset of schizophrenia and current migration**					
Before current migration	21 (80.8%)	59 (85.5%)	80 (84.2%)	–	0.545✰
After current migration	5 (19.2%)	10 (14.5%)	15 (15.8%)	
**Criminal Characteristics**					
**Criminal offense history**					
Yes	1 (3.8%)	15 (21.7%)	16 (16.8%)	–	0.061✰
No	25 (96.2%)	54 (78.3%)	79 (83.2%)		
**Offense Types**					
Violence against a person	13 (50.0%)	32 (46.4%)	45 (47.4%)	5.798	0.055
Property related	3 (11.5%)	23 (33.3%)	26 (27.4%)		
Others	10 (38.5%)	14 (20.3%)	24 (25.3%)		
**Offense location**					
Rural	6 (23.1%)	2 (2.9%)	8 (8.4%)	-	0.005✰
Urban	20 (76.9%)	67 (97.1%)	87 (91.6%)		

*SD, Standard Deviation; ✰, Fisher exact test. Δ, Others, unmarried, divorced, widowed. #, Data were missed from four people in the within-province migrant group (n = 91); °, Data were missed from three people in the within-province migrant group (n = 92)*.

**Table 6:** Comparison of the SDSS and the BPRS between inter-province migrant and within-province migrant patients.

	**Inter-provinces migrant**	**Within-provinces migrant**	**U※**	***p***
	***N* = 26**	***N* = 69**		
SDSS	6.00 (3.00, 9.00)	6.00 (3.00, 9.50)	971.50	0.532
Affect	9.00 (7.75, 11.00)	9.00 (7.00, 10.00)	717.50	0.131
Positive	11.50 (8.00, 16.00)	9.00 (8.00, 13.00)	697.00	0.093
Negative	5.00 (4.00, 6.25)	5.00 (3.50, 6.00)	852.50	0.706
Activation	4.50 (3.00, 7.00)	5.00 (3.00, 6.00)	914.50	0.881
Resistance	9.00 (7.00, 10.25)	8.00 (6.00, 10.50)	763.00	0.260
BPRS-Total	40.50 (33.75, 50.50)	39.00 (32.50, 47.00)	759.50	0.251

*※Mann-Whitney U test; Numbers outside the brackets are medians of the assessment scores, and those in the brackets are 25 and 75% values*.

**Table 7:** Comparison of demographic, clinical, and criminal characteristics between rural-to-urban migrant patients and urban-to-urban migrant patients.

	**Rural-to-urban migrant**	**Urban-to-urban migrant**	**Total**	**t/x^**2**^**	***p***
	***N* = 60**	***N* = 27**			
**Demographic Characteristics**
Age [Mean (SD)]	35.20 (9.716)	37.15 (10.439)	35.80 (9.926)	−0.845	0.400
**Gender**
Male	56 (93.3%)	23 (85.2%)	79 (90.8%)	–	0.247✰
Female	4 (6.7%)	4 (14.8%)	8 (9.2%)		
**Ethnicity**
Han	58 (96.7%)	26 (96.3%)	84 (96.6%)	–	1.000✰
Others	2 (3.3%)	1 (3.7%)	3 (3.4%)		
**Education Years#**
≤ 9	53 (91.4%)	12 (48.0%)	65 (78.3%)	19.356	<0.001
>9	5 (8.6%)	13 (52.0%)	18 (21.7%)		
**History of employment**
Yes	55 (91.7%)	26 (96.3%)	81 (93.1%)	–	0.661✰
No	5 (8.3%)	1 (3.7%)	6 (6.9%)		
**Employment status**
Employed	30 (50.0%)	11 (40.7%)	41 (47.1%)	0.641	0.423
unemployed	30 (50.0%)	16 (59.3%)	46 (52.9%)		
**Marital status**
married	22 (36.7%)	9 (33.3%)	31 (35.6%)	0.090	0.764
OthersΔ	38 (63.3%)	18 (66.7%)	56 (64.4%)		
**Living situation**
Living with relatives	34 (56.7%)	18 (66.7%)	52 (59.8%)	0.843	0.656
Living alone	19 (31.7%)	7 (25.9%)	26 (29.9%)		
Living with others	7 (11.7%)	2 (7.4%)	9 (10.3%)		
**Clinical characteristics**
Age of onset [Mean (SD)]°	28.26 (10.455)	30.22 (10.135)	28.89 (10.333)	−0.810	0.420
**History of psychiatric treatment**
Yes	44 (73.3%)	22 (81.5%)	66 (75.9%)	0.675	0.411
No	16 (26.75)	5 (18.5%)	21 (24.1%)		
**Hospitalization**
Yes	36 (60.0%)	17 (63.0%)	53 (60.9%)	0.069	0.793
No	24 (40.0%)	10 (37.0%)	34 (39.1%)		
**Illness Duration (years)**
<5	25 (41.7%)	12 (44.4%)	37 (42.5%)	0.059	0.808
≥5	35 (58.3%)	15 (55.6%)	50 (57.5%)		
**Medication at the time of offense**
Yes	12 (20%)	10 (37.0%)	22 (25.3%)	2.861	0.091
No	48 (80.0%)	17 (63.0%)	65 (74.7%)		
**Onset of schizophrenia and current migration**
Before current migration	53 (88.3%)	20 (74.1%)	73 (83.9%)	–	0.119✰
After current migration	7 (11.7%)	7 (25.9%)	14 (16.1%)		
**Criminal characteristics**
History of criminal offense
Yes	12 (20.0%)	4 (14.8%)	16 (18.4%)	0.334	0.564
No	48 (80.0%)	23 (85.2%)	71 (81.6%)		
**The type of offense**
Violence against a person	29 (48.3%)	14 (51.9%)	43 (49.4%)	1.965	0.374
Property related	19 (31.7%)	5 (18.5%)	24 (27.6%)		
others	12 (20.0%)	8 (29.6%)	20 (23.0%)		

*SD, Standard Deviation, ✰Fisher exact test. ΔOthers, unmarried, divorced, widowed. #Data were missed from two people in the rural-to-urban migrant groups and two people in the city-to-urban group(n = 83). °Data were missed from three people in the rural-to-urban migrant group(n = 84)*.

**Table 8:** Comparison of SDSS and BPRS between Rural-to-urban migrant patients and Urban-to-urban migrant patients.

	**Rural-to-urban migrant**	**Urban-to-urban migrant**	**U※**	***P***
	***N* = 60**	***N* = 27**		
SDSS	6.00 (3.00, 9.00)	5.00 (3.00, 9.00)	786.00	0.825
Affect	9.00 (7.00, 10.00)	9.00 (8.00, 11.00)	896.50	0.423
Positive	10.50 (8.00, 13.00)	9.00 (7.00, 15.00)	692.00	0.277
Negative	5.00 (4.00, 6.00)	5.00 (3.00, 6.00)	747.00	0.557
Activation	5.00 (3.00, 6.00)	4.00 (3.00, 6.00)	772.00	0.721
Resistance	8.00 (6.00, 10.75)	7.00 (6.00, 9.00)	692.50	0.278
BPRS-total	39.00 (35.00, 47.00)	34.00 (30.00, 48.00)	676.50	0.220

*※Mann-Whitney U test; Numbers outside the brackets are medians of the assessment scores, and those in the brackets are 25 and 75% values*.

## Discussion

Previous studies have widely documented the association between violence and schizophrenia (11, 28, 29). The results of this study also suggested that irrespective of whether they were migrants, schizophrenia patient crimes were more likely to involve violence against a person. However, as the victims of the non-migrant group were more likely to be killed than the victims in the migrant group, it was possible that the non-migrant patients were more violent than the migrant patients. A study in Denmark also noted that there was no reason to believe that schizophrenic immigrants were more violent than local Danish schizophrenics (12). Another explanation for this phenomenon is that most offenses committed by the migrant patients with schizophrenia in this study occurred in public places, where the victims can get timely help from others; however, most offenses committed by the local patients occurred in the privacy of their home or the middle of nowhere. There were no bystanders to intervene to stop the attacks or attempt victim rescue. Further, the non-migrant group victims were more likely to be acquaintances and relatives, whereas the victims of the migrant group were more likely to be strangers. As relatives and acquaintances may tolerate the aggressive behavior of patients with schizophrenia, they may choose not to report such behavior to the police, which means that some less severe injuries to relatives or acquaintances may not have been included in the study sample.

While the non-migrant patients mostly committed offenses against people, the migrant patients were more likely to commit property offenses, such as theft, fraud, and robbery. In line with this, some studies have also found that migration increases the level of property crime (8, 30) and has no effect on violent crime (8). A study in Denmark suggested that property-related crimes may be associated with common criminal factors (12), such as low education and a lack of occupational skills (31). It has also been suggested that the differences in labor market opportunities for different migrant groups could shape the potential impact on crime (8). The higher rate of property crimes in the migrant patient group may be resulted from economic reasons as unemployed migrant patients would be more likely to commit property-related crimes.

Most migrant patients in this study came from rural areas, which is to some extent related to the trend of rural residents migrating to urban to seek more employment opportunities after the reform and opening policy implemented in China in 1978 (32). Interestingly, it was found that more than 80% of the sample patients had suffered from schizophrenia before current migration, which suggested that many people with severe mental illness still hope to participate in society and gain some form of employment (33, 34). However, the dual stigmas of being a migrant and suffering from schizophrenia can severely reduce employment opportunities (35). Moreover, Hukou-based social exclusion, such as a lack of adequate housing and health insurance, residential segregation, and institutional barriers (36, 37), also contribute to unemployment and lack of medical resources. The current study showed that inter-provincial patients were less likely to have a history of hospitalization relative to within-province patients, which may have been because the inter-provincial migrants' health insurance did not cover them for treatment outside their native provinces (38, 39). In addition, the difference between rural-to-urban and urban-to-urban migrants only existed in the education, with a higher education level in urban-to-urban migrants, which may be attributed to the education levels of urban citizens being higher than that of rural residents in the general population (40).

The association between unemployment and crime has been widely examined in literature (41, 42), with most studies finding that property crime was the most common (41). In the current study, about 56% of the migrant patients with schizophrenia were unemployed, while up to 83% of the non-migrant patients were unemployed at the time of their offense. This result was consistent with previous studies that found most patients with schizophrenia in forensic psychiatry hospitals were unemployed at the time of their offending (12–14). Employment has the potential to prevent people from offending because it increases a person's involvement in enriching activities, allows for communication with peers, engenders a commitment to conventional life goals (43), increases self-esteem, helps develop social skills, decreases symptoms and the number of hospital admissions, and reduces stigma (44–49). Therefore, employment can significantly assist in schizophrenia patient recovery (45, 50), which in turn could reduce their chances of committing a crime. Vocational rehabilitation should be an essential target for the management of patients with schizophrenia.

The SDSS and BPRS scores (total score, and negative and positive symptom scores) for the migrant patients were lower than for the non-migrant patients, which suggested that the migrant patients may have had less severe psychiatric symptoms and less social function impairments than the non-migrant patients. The migrant patients were also younger and had suffered for a shorter time with their illnesses than the non-migrant patients, which could also explain why more migrant patients were employed than non-migrant patients (51). In China, patients with schizophrenia are generally hospitalized in the acute phase of the illness, and when the symptoms are under control, they are discharged to return to their family, that is, family members take the main responsibility for supervising and caring for people with mental disorders (52, 53). As many people with schizophrenia experience repeated recurrences after being discharged from hospital (54, 55), the most seriously ill and socially dysfunctional would be more likely to live with their families rather than migrate to look for employment.

However, regardless of the degree of illness, all patients with schizophrenia need to feel useful. Therefore, vocational rehabilitation needs to be provided for both migrant and non-migrant sufferers to ensure that they can gain and keep employment. Currently, vocational rehabilitation services in China are only at the preliminary stage (56), and more professionally trained staff and better management are needed.

This study had several limitations. First, the sample was taken from only one medical forensic assessment center in Sichuan province. As regional development impacts migration, the results cannot be generalized to forensic patients throughout China. Second, data on previous migration experiences was missing, which may have had some effect on their involvement in crime; therefore, these connections need to be examined in future studies. Third, the sample size in subgroups was relatively small, especially in the inter-provinces migrant group, which may have limited the statistical ability to detect slight differences between the groups. Future studies with larger subgroup sample size are needed to verify the results of the present study. Fourth, this study was a retrospective study based on archival research, which limited the potential for checking data accuracy and thorough comprehensiveness of the extracted data. Fifth, since the data was based on a sample of suspects rather than convicts, the findings on associations with criminal conduct may not be conclusive since those who are charged with some crimes may not eventually be convicted for them. This could potentially alter the descriptive patterns and attendant associations. Sixth, the application of the SDSS and BPRS to archival contents could potentially under- or over-rate the presence of items on both scales since not all items may be fully captured in the archival content.

## Conclusion

In spite of the aforementioned limitations, the data presented here suggests that the migrant patients with schizophrenia, are younger, and have less severity of psychiatric symptoms with better social function, than non-migrant patients. The higher rate of property crimes in the migrant patients may result from the failure in employment and the subsequent economic difficulties. These results indicate that some patients with schizophrenia still preserved the motivation and willingness of social and vocational participation by migration. Employment is beneficial to improve not only economic situation, but also social function. However, many social obstacles may preclude them from participating in social activities and getting employed. Thus, policies focusing on vocational rehabilitation and developing more appropriate employment for patients with schizophrenia, especially for migrant patients are crucial for reducing their chances of committing a crime. Moreover, efforts to reduce stigma and residential segregation, and promote welfare in health insurance and housing could also aid in the recovery of the patients and the reduction of criminality. Future studies with larger sample size to further investigate the interaction between schizophrenia, migration and criminality are warranted.

## Data Availability Statement

The raw data supporting the conclusions of this article will be made available by the authors, without undue reservation.

## Ethics Statement

The studies involving human participants were reviewed and approved by Medical Ethical Committee of Sichuan University. Written informed consent for participation was not required for this study in accordance with the national legislation and the institutional requirements.

## Author Contributions

ZH, GL, and YH conceived and designed the study. ZH and GL provided oversight and direction. YH, YL, and SW contributed to data extracting. YH and YG contributed to data analysis and interpretation. YH drafted the manuscript. YH, ZH, GL, YG, SW, and YL contributed to the revision of the manuscript. All authors contributed to the article and approved the submitted version.

## Funding

This work was supported by China Postdoctoral Science Foundation [grant number 2018M643488] and the National Natural Science Foundation of China [grant number 81901928].

## Conflict of Interest

The authors declare that the research was conducted in the absence of any commercial or financial relationships that could be construed as a potential conflict of interest.

## Publisher's Note

All claims expressed in this article are solely those of the authors and do not necessarily represent those of their affiliated organizations, or those of the publisher, the editors and the reviewers. Any product that may be evaluated in this article, or claim that may be made by its manufacturer, is not guaranteed or endorsed by the publisher.
